# Vaccination as an alternative to non-drug interventions to prevent local resurgence of COVID-19

**DOI:** 10.1186/s40249-022-00960-6

**Published:** 2022-03-26

**Authors:** Jinhua Pan, Wenlong Zhu, Jie Tian, Zhixi Liu, Ao Xu, Ye Yao, Weibing Wang

**Affiliations:** 1 School of Public Health, Key Lab of Public Health Safety of the Ministry of Education, Fudan University, Shanghai, 200032 China; 2 Department of Epidemiology, School of Public Health, Fudan University, Shanghai, 200032 China; 3 Shanghai Institute of Infectious Disease and Biosecurity, School of Public Health, Fudan University, Shanghai, 200032 China; 4 Department of Biostatics, School of Public Health, Fudan University, Shanghai, 200032 China

**Keywords:** COVID-19, Vaccine, Non-pharmaceutical interventions, Compartment model, Monte Carlo simulation

## Abstract

**Background:**

While a COVID-19 vaccine protects people from serious illness and death, it remains a concern when and how to lift the high-cost and strict non-pharmaceutical interventions (NPIs). This study examined the joint effect of vaccine coverage and NPIs on the control of local and sporadic resurgence of COVID-19 cases.

**Methods:**

Between July 2021 and January 2022, we collected the large-scale testing information and case number of imported COVID-19 patients from the website of the National Health Commission of China. A compartment model was developed to identify the level of vaccine coverage that would allow safe relaxation of NPIs, and vaccination strategies that can best achieve this level of coverage. We applied Monte Carlo simulation 50 000 times to remove random fluctuation effects and obtain fitted/predicted epidemic curve based on various parameters with 95% confidence interval at each time point.

**Results:**

We found that a vaccination coverage of 50.4% was needed for the safe relaxation of NPIs, if the vaccine effectiveness was 79.3%. The total number of incidence cases under the key groups firstly strategy was 10^3^ times higher than that of accelerated vaccination strategy. It needed 35 months to fully relax NPIs if the key groups firstly strategy was implemented, and 27 months were needed with the accelerated vaccination strategy. If combined the two strategies, only 8 months are needed to achieve the vaccine coverage threshold for the fully relaxation of NPIs. Sensitivity analyses results shown that the higher the transmission rate of the virus and the lower annual vaccine supply, the more difficult the epidemic could be under control. When the transmission rate increased 25% or the vaccination effectiveness rate decreased 20%, 33 months were needed to reduce the number of total incidence cases below 1000.

**Conclusions:**

As vaccine coverage improves, the NPIs can be gradually relaxed. Until that threshold is reached, however, strict NPIs are still needed to control the epidemic. The more transmissible SARS-CoV-2 variant led to higher resurgence probability, which indicates the importance of accelerated vaccination and achieving the vaccine coverage earlier.

**Graphical Abstract:**

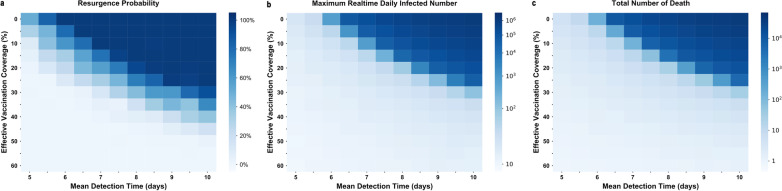

**Supplementary Information:**

The online version contains supplementary material available at 10.1186/s40249-022-00960-6.

## Background

On March 11, 2020, the World Health Organization (WHO) characterized Coronavirus disease 2019 (COVID-19) as a pandemic [1]. As of 25 February 2022, 228 countries/regions have reported at least one case of COVID-19 infection [2]. Due to the lack of effective drugs and vaccines at that time, non-pharmaceutical interventions (NPIs) played a significant early role in control of the COVID-19 outbreak in China and other countries [3–6]. However, long-term and strict NPIs can lead to significant economic and social costs, and new infections could emerge if they are relaxed too early [7–9].

COVID-19 was under control in China at the end of March 2020, but China faced a new challenge of re-contamination due to close contact with foreign visitors because of increased travel to China [10, 11]. The National Health Commission of China recommended that foreign personnel should be subject to nucleic acid testing and centralized quarantine or household quarantine for 14 days upon entry. They also recommended that foreigners with confirmed or suspected COVID-19 identified in local customs quarantine should receive treatment in designated hospitals [12]. However, from the end of March 2020 there were some sporadic and local outbreaks in the regions of Suifenhe, Harbin [13], Shulan [14], Beijing [15], Urumqi [16], Dalian [16], Qingdao [17], Kashgar, Chengdu [18], and Shanghai [19]. Some of these outbreaks were related to imported cases [20, 21] and others to cold-chain foods [22, 23]. Quarantine, treatment of confirmed cases, comprehensive close contact tracking, large-scale nucleic acid detection, and community lockdown are NPIs that have greatly prevented COVID-19 transmission and helped to control local outbreaks [24–28]. Although these measures are effective, they are also costly and disruptive [29].

An effective vaccine is considered the key for preventing further morbidity and mortality from COVID-19 [30]. As of May 28, 2021, there were 184 vaccine candidates in pre-clinical development, and 102 candidate vaccines currently undergoing clinical trials worldwide [31]. By the end of 2021, the total global production capacity of the 12 currently approved COVID-19 vaccines was estimated to reach about 10 billion doses [32]. As of December 31, 2020, 14 COVID-19 vaccines developed in China were undergoing clinical trials, including 5 in phase III trials. The National Medical Products Administration granted conditional approval for first COVID-19 vaccine in China on December 30, 2020 [33]. China now provides COVID-19 vaccines free-of-charge to all populations [34]. The vaccination strategy of China gives priority to those with high risk (including doctors and individuals engaged in the import of cold chain foods, public transportation, etc.), followed by eligible members of the general population [35]. China had a production capacity of 610 million doses in 2020 and was projected to produce at least one billion doses by the end of 2021 [36]. However, China’s large and heterogeneous population has made it difficult to achieve herd immunity in a short time. Thus, NPIs are still important for the prevention and control of imported COVID-19 cases and local outbreaks.

There were several studies that analyzed the joint effects of vaccines and NPIs in controlling the pandemic [37–40]. Before sufficient effective vaccine coverage reached, relaxation of NPIs would cause a new wave of infections. However, most of the current studies focused on the scenario that there had been a COVID-19 outbreak or epidemic, and none have assessed the impact of vaccination and NPIs in avoiding a resurgence in a city or country. In addition, few studies have considered the impact of variants due to increased transmissibility and attenuated effectiveness of vaccine in models [37, 39].

In this study, we developed a compartment model to determine the role of vaccination and NPIs on avoiding COVID-19 resurgence at the city level. We focused on the relationship of vaccine coverage with the timing and conditions of the relaxation of different NPIs.

## Methods

**Fig. 1:**
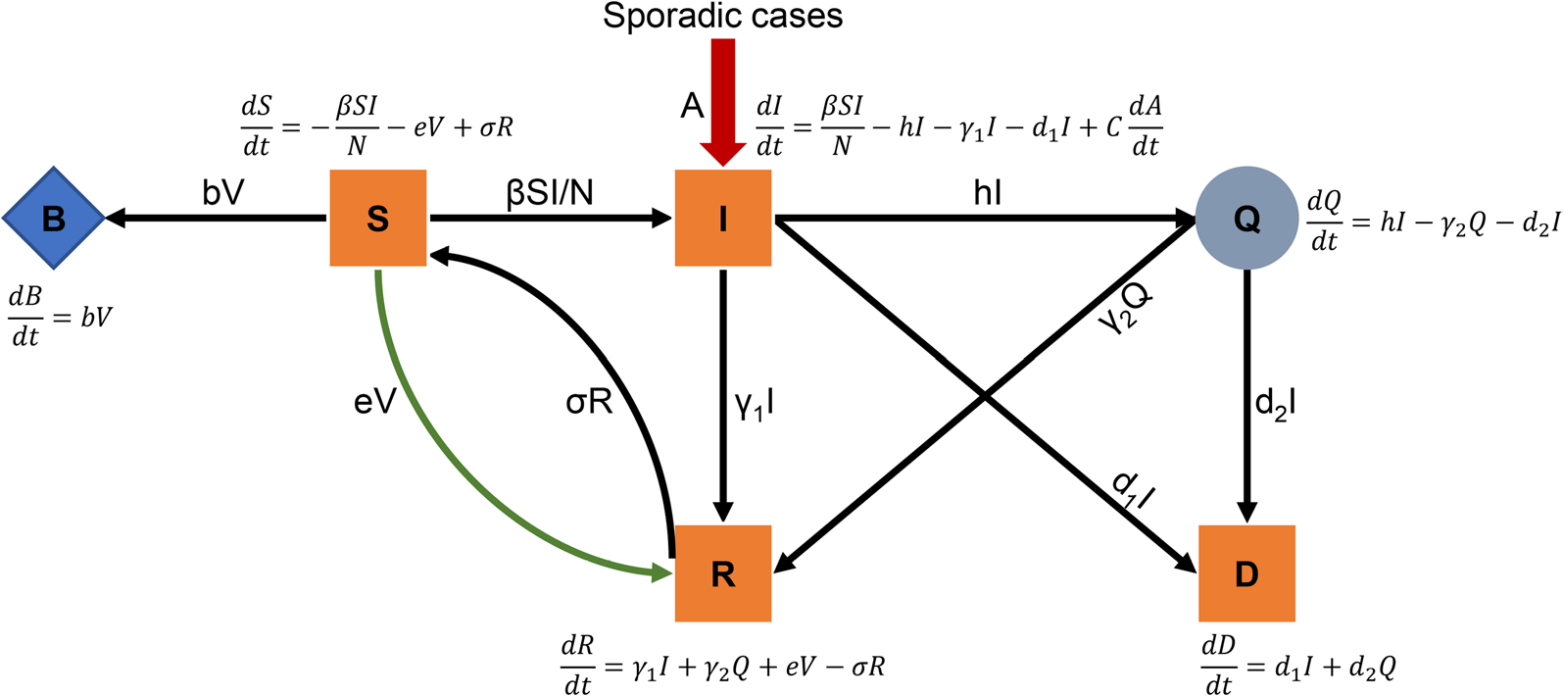
The BSIQDRS model. We extended the classic SIRS model to include six compartments: susceptible (S), adverse reactions (B), infectious (I), quarantined cases (Q), removed (R), and deaths due to COVID-19 (D). Each compartment attached an equation in the model

A model of SARS-CoV-2 transmission, with the population of a city stratified into six compartments, was developed (Fig. 1). The model city had a population size similar to Beijing and demographics similar to the general population of China, although the results are generalizable to other populations. The model assumed that vaccine-induced immunity lasted at least 2 years (model time horizon).

### Data collection

Non-pharmaceutical interventions (NPIs) refer specifically to government interventions to control the spread of COVID-19 that do not use drugs, such as school closures, masks and large-scale nucleic acid testing [41]. Large-scale test data were from the website of the National Health Commission (http://www.nhc.gov.cn/), which had data on sporadic cases in Beijing [42], Dalian [43], Qingdao [17], Chengdu [18], and Shanghai [19]. These data were averaged as the estimation of sporadic cases in the model.

### The compartmental model

The model analyzed a population stratified into six compartments by extension of the classic SIRS model [44] to a BSIQDRS model (Fig. 1). The BSIQDRS model is an abbreviation for each compartment in the model, and each letter in the name of BSIQDRS model represents the corresponding compartment. This model incorporates three additional compartments to account for individuals with adverse reactions (B), quarantined cases (Q) and deaths due to COVID-19(D) (where S: susceptible people, I: infected people, R: recovered people, and N: total population (S + I + Q + R)). Dynamics of these compartments across time t was described in Fig. 1. The differential equations of dynamic model as following.

$$\frac{dS}{dt}=-\frac{\beta SI}{N}-eV+\sigma R$$

$$\frac{dI}{dt}=\frac{\beta SI}{N}-hI-{\gamma }_{1}I-{d}_{1}I+C\frac{dA}{dt}$$

$$\frac{dQ}{dt}=hI-{\gamma }_{2}Q-{d}_{2}Q$$

$$\frac{dR}{dt}={\gamma }_{1}I+{\gamma }_{2}Q+eV-\sigma R$$

$$\frac{dB}{dt}=bV$$

$$\frac{dD}{dt}={d}_{1}I+{d}_{2}Q$$

In addition, a represented a Poisson process to simulate sporadic non-local COVID-19 arrival time with a parameter C (number of patients selected randomly from a discrete uniform distribution for each time). Furthermore, β was the transmission rate of COVID-19 cases, defined as the average number of individuals that a case can infect per day [11], V was the daily vaccination number, e was the vaccine effectiveness, ${\upsigma }^{-1}$ was the immunity duration, h was the rate of an infectious cases to isolated cases (${h}^{-1}$: actual infectious period), ${\gamma }_{1}^{-1}$ and ${\gamma }_{2}^{-1}$ were the rates of patients recovered, d_1_ and d_2_ were case fatality rates, and b was the adverse reaction rate. Additionally, β was a time-varying coefficient which was simulated as the quotient of randomly selected basic reproduction number and mean infectious period without quarantine. Parameters e, V, σ, h, γ_1_, γ_2_, d_1_, d_2_ and b were time-varying coefficients (fixed constant with 10% perturbation).

### Monte Carlo simulations

In this study, we made efforts to do numerical analysis on infectious disease stochastic modelling. In addition, since stochastic process were included in the model and most of parameters were time-varying and time-dependent, we applied Monte Carlo simulations 50 000 times to remove random fluctuation effects and obtain fitted/predicted epidemic curve based on various parameters with 95% confidence interval (95% *CI*) at each time point.

### Parameter settings and initial states

Parameter settings for the main analysis were summarized in Additional file 1: Table S1. The BSIQDRS infectious disease stochastic model was used to simulate three-year results in this study. We set β = R_0_/τ according to [45, 46], where *R*_0_ was basic reproduction number which varied around 2.5 [47, 48]. τ was mean infectious time, with an estimated 9 days according to previous papers reported [49, 50]. We set vaccine effectiveness e = 0.7934 according to the results of phase III clinical trials of the vaccine in China [51, 52]. The transmission rate of the population reduced to 0 as they effectively protected by vaccination. β (or *R*_0_) was still the initial value, for those who were not vaccinated or who were vaccinated but not effectively protected. We set up two vaccination scenarios. In addition, this study assumed that 10% of the people in the city were the key population (held higher chance to contact foreign imported patients and had higher transmission rate). We compared simulation results based on key group vaccinated firstly with slow situations in this article. We assumed that vaccine-induced immunity last for around two years (our time horizon), thus the σ equal to 1/730. *h* was the rate for an infectious cases becoming isolation (mean detection time, MDT), which refers to both pharmacological and NPIs, including but not limited to large-scale nucleic acid testing, which can reduce the detection time of patients. We used *h* to represent intensity of the NPIs. The average incubation period of COVID-19 is around 5.18 days [53] Since COVID-19 can hardly be detected in the first several days until onset [48], we assumed that the most stringent NPIs were detected on day 5, with a mean detection time, ${h}^{-1}$ of 5 days. Additionally, the average duration from symptom onset to isolation was 4.1 ± 3.7 days [54], and thus, the largest mean detection time, *h*, should be around 10 days. We assumed that infected cases in this city would recover 14 days after infections, and ${\gamma }_{1}^{-1}$ and ${\gamma }_{2}^{-1}$ were 14. We assumed the infectious cases and the quarantined cases shared the same case fatality rate in this city, and we set d_1_ and d_2_ = 0.005/21, which were similar to the city like Shanghai [19], Beijing [42] and Qingdao [17]. A was represented as a Poisson process to simulate sporadic foreign imported COVID-19 arrival times with a parameter C, which is the number of patients selected randomly from a discrete uniform distribution (1–7 patients) each time. We assumed the frequency of sporadic cases in this city is similar to that in Beijing, China, which was around 5 times from September to December, 2020 in 120 days.

### Estimated scenarios in which vaccines can replace current NPIs

The stochastic simulations described above were used to estimate the daily number of infected cases, death rate, and probability of resurgence under different scenarios. First, vaccine coverage was assumed to range from 0 to 60% of the total population in 5% increments (the proportion in compartment R). In this analysis, b represented the daily vaccination rate, assuming that after a certain vaccination coverage is reached, the rate of vaccination is consistent with the rate of vaccine failure. Second, to model different levels of nucleic acid detection, we used mean detection time (*h*^−1^) to represent the intensity of nucleic acid detection, and defined *h*^−1^ as the actual infectious period. It was assumed that *h*^−1^ varied from 5 to 10 days in 0.5 day increments; an *h*^−1^ of 5 days meant that the person was identified and isolated on the first day of symptom onset [48]. An individual is infectious during the four days before symptom onset, but nucleic acid testing is insufficiently sensitive during this time due to the low viral titer, and it represents the NPIs were not rigorous when *h*^−1^ was 10 days. There were 143 combinations of parameters and each combination runs Monte Carlo simulation for 50 000 times.

### Predicting the risk of resurgence after cessation of NPIs

The risks of halting NPIs on the probability of resurgence, daily number of infections, and the number of deaths were determined. This included relaxing all NPIs at t days after the first day of vaccination. Time to resurgence was defined as the number of days from lifting controls to when the number of active cases above seven patients in some day (detailed sensitivity analysis for resurgence threshold can be seen in Additional file 1: Fig. S1). It was assumed necessary to consider NPIs and vaccination rates when the resurgence probability exceeded 20%.

We performed Monte Carlo simulations for 50 000 times with different parameter combinations. The probability of resurgence was the proportion of simulations in which a resurgence occurred, and we simulated the following three years from the day of vaccination. Several vaccination scenarios were considered. First, it was assumed that 5 million doses of vaccines were available every year, and the city could choose to vaccinate high-risk individuals or to vaccinate everyone (no priorities). Second, it was assumed the government could provide 16 million vaccine doses in the first year and 2 million doses in each subsequent year, and the city could choose to vaccinate high-risk individuals or to vaccinate everyone (no priorities). Then, these two plans were combined to create four different vaccination scenarios. The slow vaccination plan was that the city had an annual supply of 5 million vaccine doses and the city chose to vaccinate everyone (no priorities). High-risk individuals were workers may be exposed to dangerous occupation of COVID-19, such as workers in the Centers for Disease Control and Prevention (CDC), hospital workers, delivery workers, cold food chain workers, and so on. This study assumed high-risk individuals accounted for 10% of the total population of the city.

### Sensitivity analyses for the real data

We designed five sensitivity analyses to test the robustness of our results from real data. For each of the sensitivity analyses, we fixed parameters and initial states to be the same as the main analysis except for those mentioned below. According to COVID-19 Weekly Epidemiological Update Reports Edition 43, published 8 June 2021 of World Health Organization (WHO) [55], some variants of SARS-CoV-2 have resulted in changes in transmissibility, for instance, Alpha (B.1.1.7) variant first detected in United Kingdom may increase 45–71% transmissibility, Beta (B.1.351) variant first detected in South Africa may increase 50% transmissibility, and the transmissibility of Gamma (P.1) variant which first detected in Brazil may be 1.4–2.2 times as the original transmissibility of SARS-CoV-2. Therefore, for analysis (S1), we set different scenarios of transmission rate of SARS-CoV-2 under the slow vaccination strategy. The different cases of transmission rate settings are assumed to be how many times the transmission rate is increased from the original basis, respectively 0, 25, 50, 75 and 100%. For analysis (S2), we set the different total number of vaccinations scenarios per year: 3 million, 4 million, 5 million, 6 million, 7 million. For analysis (S3), effective vaccine rate was assumed to range from 60 to 100% of the total population in 10% increments. For analysis (S4), we set different vaccination effectiveness times scenarios: 1 year, 1.5, 2, 2.5, 3 years (2 years was our baseline scenario in the main analysis). For analysis (S5), we assume this city have 30, 60, 90, 120, 150 sporadic foreign imported COVID-19 cases per year (60 sporadic foreign imported COVID-19 cases per year was our baseline scenario in the main analysis).

## Results

### Scenarios in which vaccines can lead to relaxation of NPIs

**Fig. 2:**
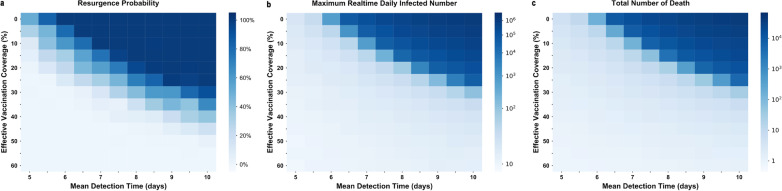
Joint effects of NPIs and vaccination on resurgence probability (**a**), daily infections (**b**), and total deaths (**c**). For each plot, each row represents a different NPIs intensity (mean detection time) and each column represents effective vaccination coverage (percentage of the total population to be vaccinated multiply vaccine effective rate). Colors represent the probability of resurgence / maximum daily infected number / total death number, ranging from 0 (white) to 100%/10^6^/10^5^ (blue)

We assumed the mean detection time was 5 days and the vaccination rate was 0% (strictest NPIs without vaccination) as a basic scenario because of the low probability of an outbreak (resurgence probability: 51%, Fig. 2a). This meant that when the vaccine introduced in this scenario, we can relax the NPIs gradually. If the vaccination rate was 10% (vaccine coverage: 12.6%), prolonging the detection time to 5.5 days led to the same outcome. The same outcome also occurred if the vaccination rate was 20% (vaccine coverage: 25.2%) and the detection time was more than 6.5 days; if the vaccination rate was 30% (vaccine coverage: 37.8%) and the detection time was more than 8 days; and if the vaccination rate was 40% (vaccine coverage: 50.4%) and the detection time was more than 10 days. Thus, when vaccine coverage reaches 50.4%, no matter whether the large-scale detection was strict or not, we can obtain the same effect of basis scenario (because we can prolong the detection time to longest 10 days), which means we can fully lift the NPIs.

During the two-year simulation period, nder the same intervention intensity, the probability of resurgence declined as vaccine coverage increased. When effective vaccination coverage was 40–60%, the probability of resurgence was low regardless of the extent of detection. When effective vaccination coverage was 40%, NPIs could be relaxed (i.e., mean time interval from infection to isolation was longer), and the resurgence probability was the same as when there were strict NPIs without vaccination (resurgence probability: 51%). Strict NPIs were still needed when the vaccine coverage was 10–30%. If there was no vaccine, there was a high probability of resurgence if NPIs were relaxed. We observed the same trends in terms of maximum daily infected cases and deaths (Fig. 2b, c). In particular, when effective vaccine coverage was less than 40%, the daily number of infected cases and deaths increased exponentially as the NPIs were relaxed; when 40% of the population was vaccinated (vaccine coverage: 50.4%), NPIs may be safely relaxed.

### Predicting the risk of resurgence after relaxation of NPIs

**Fig. 3:**
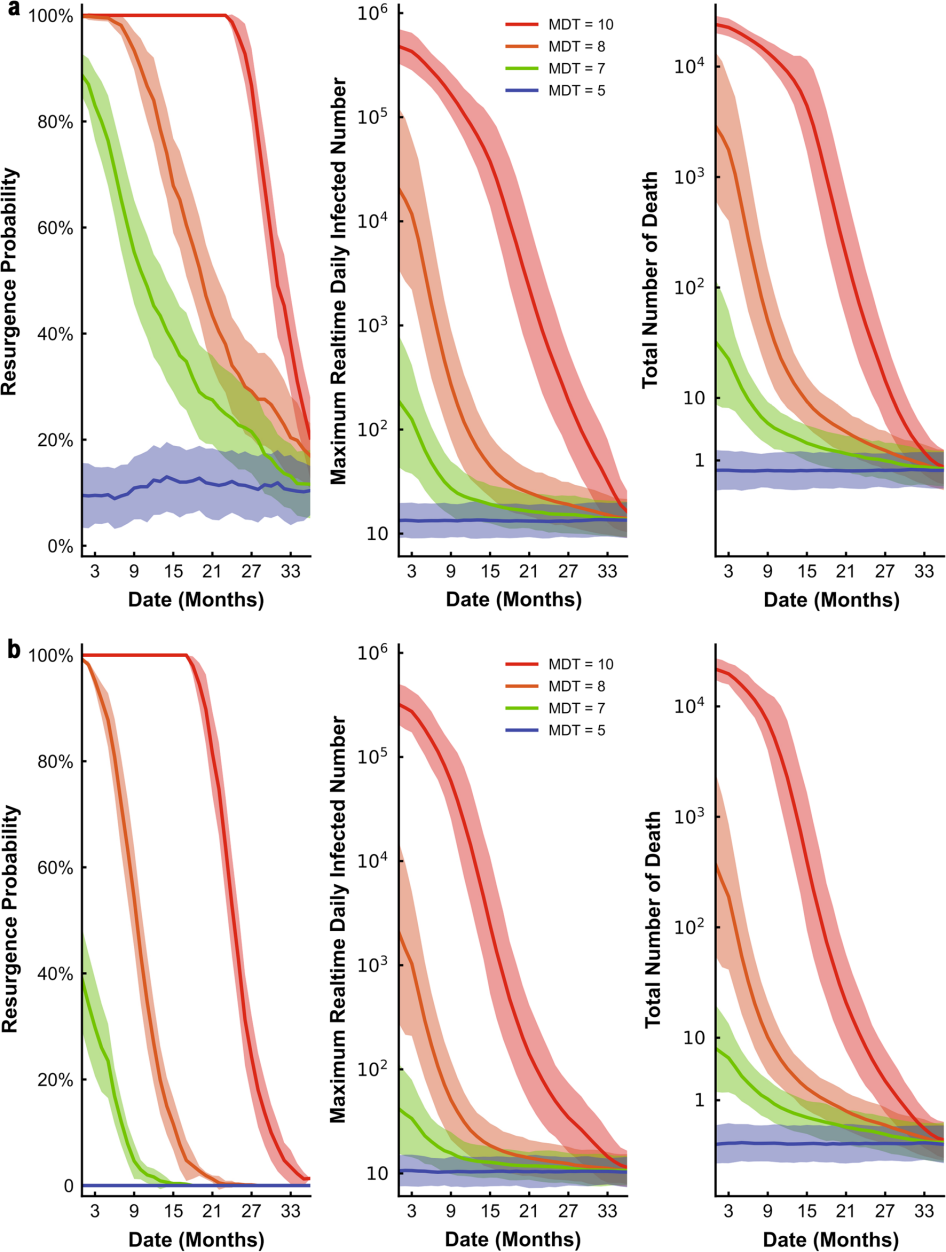
Effect of relaxing NPIs under no priority vaccination strategy **a** and high-risk population first vaccination strategy **b** on resurgence probability, daily infections, and total deaths when there were 5 million vaccine doses per year. Colors represent different NPIs intensity (mean detection time), ranging from 5 (purple, “baseline”) to 10 (red). For each plot, each row represents the date to lift NPIs (months, from the day of vaccination) and each column represents resurgence probability, daily infections, and total deaths. The line represents resurgence probability, daily infections, and total deaths when lifting NPIs, the shaded area represents averaged results of MC simulations with 95% confidence interval

We assessed the effect of relaxing NPIs under no prior vaccination strategy (Fig. 3a) and high-risk population first vaccination strategy (Fig. 3b) on resurgence probability, daily infections, and the total number of deaths when there were 5 million vaccine doses per year. Under these scenarios, with the most relaxed NPIs (MDT = 10) and regardless of vaccination strategy, there was a high initial probability of resurgence and this continued over time. For the vaccination strategy with no prioritized individuals, a continuing relaxation of NPIs, and a continuous increase of vaccination, the probability of resurgence was still high. Only after 27 months, when vaccination coverage reached 26.1%, was there a decline in the probability of resurgence (Fig. 3a). When high-risk individuals had priority for vaccination, this time could be shorter, and the effect of the vaccine was evident after 21 months when the NPIs were most relaxed (Fig. 3b). If NPIs were in place (MDT = 5/7/8) at the time of vaccination, the probability of resurgence, the number of infections, and the number of deaths decreased in the early stage. Moreover, stricter interventions led to a sharper decline. After the third year of vaccination, the most relaxed NPIs (MDT = 10) was also associated with a probability of resurgence that was less than 20%.

**Fig. 4:**
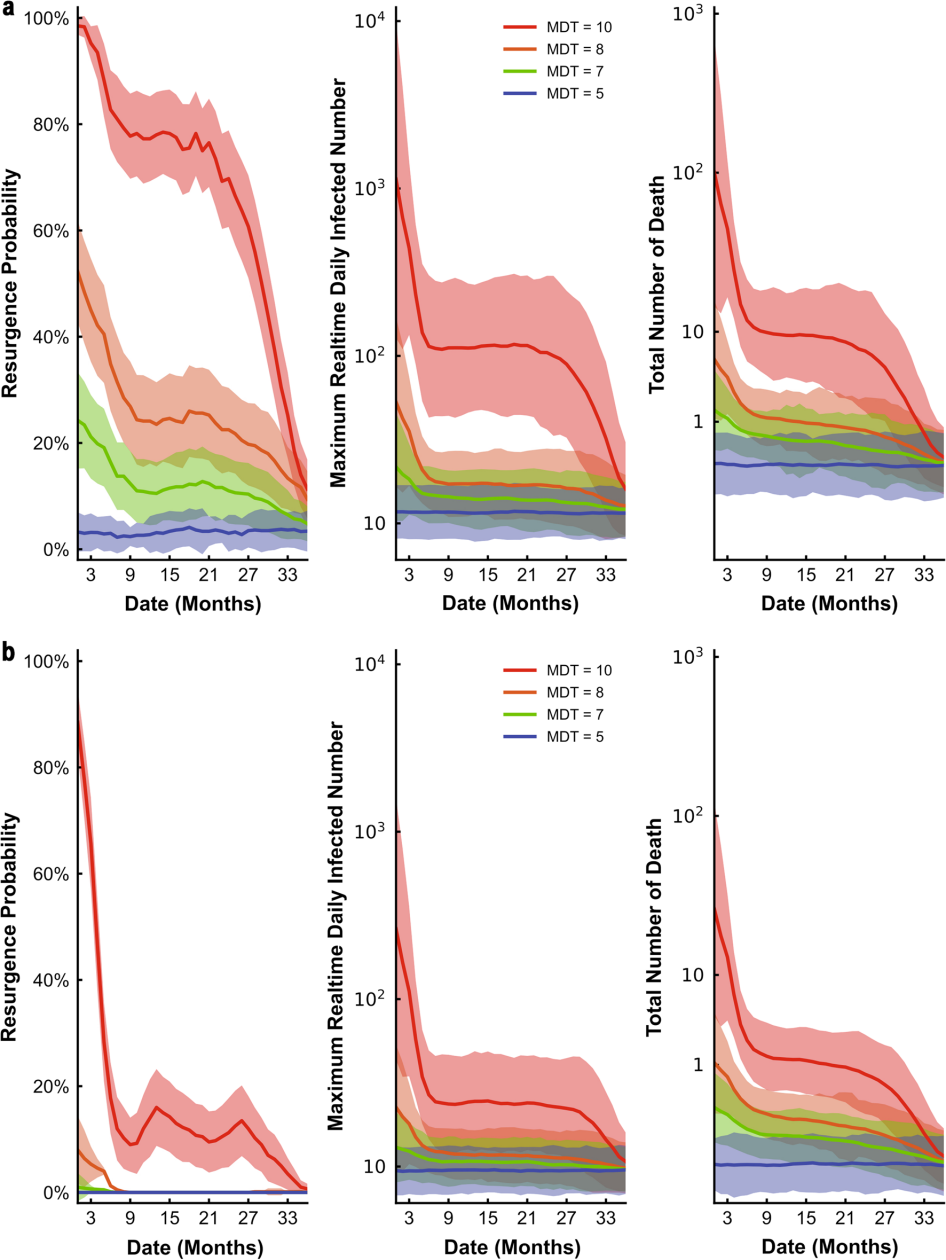
Effect of relaxing NPIs under no priority vaccination strategy **a** and high-risk population first vaccination strategy **b** on resurgence probability, daily infections, and total deaths when there were 16 million vaccine doses during the first year. Colors represent different NPIs intensity (mean detection time), ranging from 5 (purple, “baseline”) to 10 (red). For each plot, each row represents the date to lift NPIs (months, from the day of vaccination) and each column represents resurgence probability, daily infections, and total deaths. The line represents resurgence probability, daily infections, and total deaths when lifting NPIs, the shaded area represents averaged results of MC simulations with 95% confidence interval

We also assessed the effect of relaxing NPIs under ‘no priority’ vaccination strategy (Fig. 4a) and high-risk population first vaccination strategy (Fig. 4b) on resurgence probability, daily infections, and total deaths when there were 16 million vaccine doses in the first year and 2 million doses in the following two years. In these cases, even if when the MDT was 10 days, the probability of resurgence gradually declined, and the effect of vaccination appeared during the first month (Fig. 4a, b). In addition, for the same intervention intensity, the risk of resurgence is lower for the high-risk population first vaccination strategy than for the no priority vaccination strategy. With sufficient vaccine supply, high-risk population first vaccination strategy was more effective to reduce the resurgence probability at the early stage, even with the greatest relaxation of NPIs. For high-risk population first vaccination strategy, after 9 months, the risk of resurgence was less than 20% (Fig. 4b). If slightly intensified NPIs were implemented at this time, the epidemic was controlled (Fig. 4b).

**Fig. 5:**
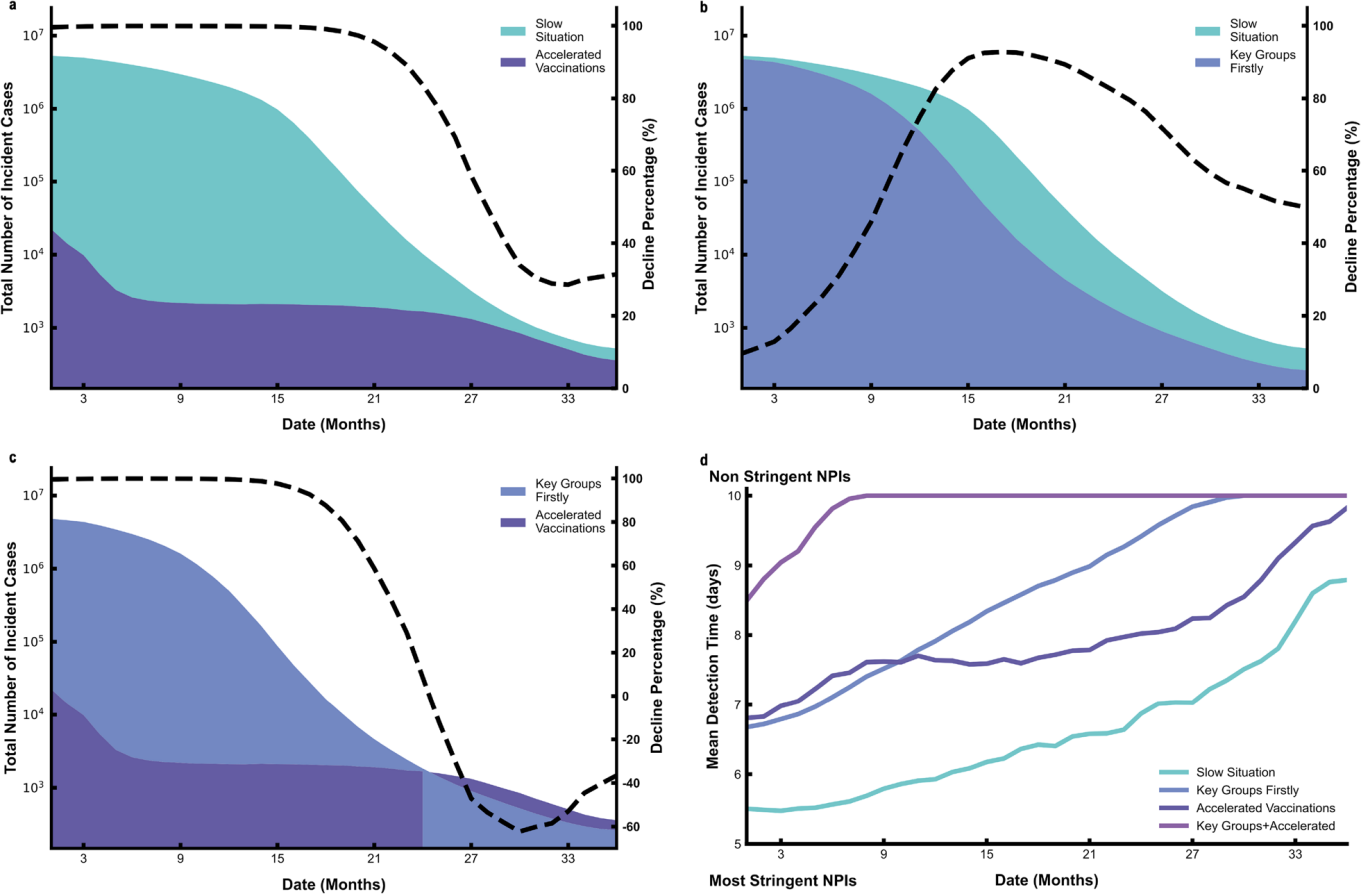
Comparison of different vaccination strategies. For each plot, each row represents the date to lift NPIs (months, from the day of vaccination) and each column represents total incidence number **a**–**c**, and different NPIs intensity (**d**). Colors represent different vaccination strategies. The dashed line in a, b and c represents the decline percentage (%) between two strategies

We then compared the effects of multiple strategies (Fig. 5). During the first 21 months, there were far fewer COVID-19 cases in the accelerated vaccination scenario than the slow vaccination scenario, the decline percentage were nearly 100% (Fig. 5a). This indicated that the impact of rapid mass vaccination was very significant. We also determined the effect of prioritizing vaccines for high-risk individuals when 5 million doses were available (Fig. 5b), the number of COVID-19 cases was reduced by about 60% over three years These results indicated that, the targeting of COVID-19 vaccinations provides an important benefit. Finally, prioritizing COVID-19 vaccinations for high-risk individuals and accelerating vaccination led to a very dramatic decline in total incidence (Fig. 5c). We then assessed that how long can we lift all NPIs (Fig. 5.d). We estimated that 8 months are needed to achieve the vaccine coverage threshold for the full relaxation of NPIs in the combination strategy of accelerated vaccination and key groups firstly. However, if we conduct a slow vaccination strategy, NPIs would not be fully liberalized in three years (Fig. 5.d).

### Sensitivity analyses

**Fig. 6:**
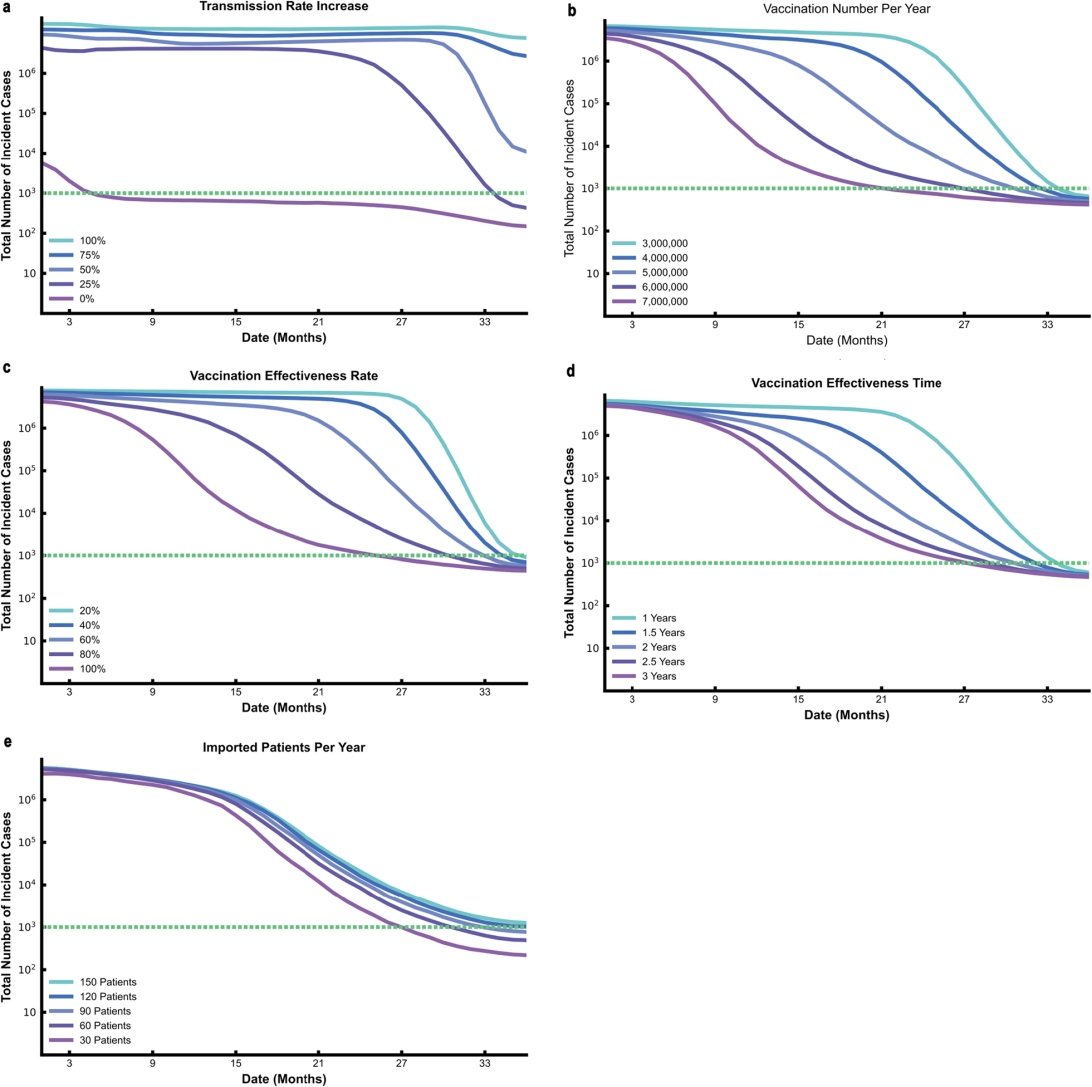
Sensitivity analyses on transmission rate (**a**), vaccination number per year (**b**), vaccination effectiveness rate (**c**), vaccination effectiveness time **d** and imported patients per year (**e**). For each plot, each row represents the date to lift NPIs (months, from the day of vaccination) and each column represents total incidence number. Colors represent different scenarios of every parameter. Horizontal dotted line in each plot represents the threshold of total incidence number (1000 cases)

We performed a series of sensitivity analyses to test the robustness of our results by varying the transmission rate, the number of vaccinations per year, the vaccination effectiveness rate, the lengths of vaccination effective time and the imported patients per year. Sensitivity analyses results shown that the total incidence number were negatively correlated with the vaccination number per year, vaccination effectiveness rate, the specified vaccination effective time, while the total incidence number were positively correlated with the number of imported patients per year, and transmission rate (Fig. 6.). If the transmission rate of the virus increased by 25%, the total number of the incidence cases was 750 times than that of the baseline transmission rate, and it would take an additional 30 months to reduce the incidence cases less than 1000 (Fig. 6a). When the vaccine supply reaches 7 million doses per year, the total incidence number is significantly less than that when the vaccine supply is 3 million doses per year, and 32.9% patients can be reduced in three years in that scenario (Fig. 6b). In addition, we found that vaccination effectiveness rate and vaccine duration had less impact on the total number of cases compared to the number of vaccinations (Fig. 6c, d). Imported patients had few influences on the total number of patients (Fig. 6e). With the increasing of vaccination number per year, effective vaccination rate and the vaccination effectiveness time, the resurgence probability decreased (Additional file 1: Fig. S2).

## Discussion

The major result of this modeling study is that gradual relaxation of NPIs, such as large-scale detection and quarantine, would be safe as vaccine coverage increased. In particular, for the transmission of wild strain in a city with a population of 20 million, NPIs can be relaxed when vaccine coverage reaches 50.42%. The outcomes will be improved if the vaccination strategy was accelerated or high-risk groups were given priority.

Consistent with previous studies [37, 38, 40], our results suggested that the relaxation of NPIs before establishment of sufficient immunity increased the probability of COVID-19 resurgence (maximum daily infected cases and the number of deaths). In particular, our model indicated that if vaccine effectiveness was 79.3%, vaccine coverage must be 50.4% before NPIs can be fully relaxed, and when coverage reached 75.6%, resurgence was very unlikely. The vaccination coverage threshold 50.4% was estimated based on the transmissibility of the wild strain and the vaccine efficacy against wild strain infection. The presence of many mutations in the SARS-CoV-2 spike (S) protein region of the Omicron (B.1.1.529) variant has led to a high rate of transmission and decreased disease severity after the Alpha (B.1.1.7), Beta (B.1.351), Gamma (P.1), and Delta (B.1.617), the protection from existed COVID-19 vaccines seems to be attenuated against the disease [55]. As our results suggested that with the increasing of transmissibility of variants and the possible decreasing of vaccine effectiveness, the vaccine coverage threshold for safely relaxing NPIs would be higher. In the presence of highly transmissible variants, strict NPIs were also needed to avoid resurgence before reaching a sufficient vaccine coverage [37, 39]. The more transmissible the new variants would be, the faster speed of vaccination are needed.

A localized COVID-19 outbreak occurred in Guangzhou on May 21, 2021 and on May 31, 2021, there were 34 symptomatic cases and 8 asymptomatic infections [56]. According to public data, we estimated that vaccine coverage was 40% (effective vaccinated population: 31.74%) in Guangzhou on May 23, 2021. If Guangzhou implemented moderate NPIs, in particular if the MDT was 7 to 8 days, the probability of resurgence was 30% to 60%. This suggested that NPIs, such as the social distancing, large-scale nucleic acid testing, close contact tracking, and centralized isolation, still played a significant role in reducing the probability of resurgence and controlling local resurgences before the vaccine coverage threshold was attained. On 29 May 2021, there were 12 COVID-19 asymptomatic infections in Guangzhou [56], which had met the criteria of resurgence. However, even if Guangzhou lifted all NPIs, the resurgence probability was estimated by 90%. Those facts suggested that the virus might be more infectious, or the efficiency of vaccines may be not high enough for the variants. As vaccine coverage increased, the NPIs can be gradually relaxed without increasing the risk of a resurgence.

We estimated that 8 months are needed to achieve the effective vaccine coverage threshold (50.4%) for the fully relaxation of NPIs in the combination of accelerated vaccination strategy and high risk groups first strategy. However, if a slow vaccination strategy was conducted, NPIs would not be fully liberalized in three years. These results suggested that acceleration of vaccination and targeting high-risk groups could reduce the probability of COVID-19 resurgence, especially when implemented early during the vaccination program [37, 38, 57]. Accelerating vaccination is also necessary to prevent the transmission and spread of more contagious SARS-CoV-2 variants [57]. Compared with no vaccination, introducing vaccination had high cost-effectiveness [58].

Vaccine hesitancy is a complex public health issue, and obviously hinders vaccination programs. At the end of March 2020, when the first wave of the COVID-19 outbreak was controlled in China, 67.1% to 91.3% of people were willing to accept the available COVID-19 vaccine [59, 60]. However, in May 2020, 83.5% of people said they had the intent intended to get vaccinated in China, and only 28.7% reported they definitely intended to get vaccinated [61]. Because of the successful control of COVID-19 outbreaks and the low incidence rate of COVID-19 in China, many people believed that vaccination was unnecessary [59]. Our results suggested that there is a high probability of resurgence if the NPIs are relaxed before the target vaccine coverage is achieved. Therefore, to reduce vaccine hesitancy, it is necessary to educate the general public about the safety, benefits, and importance of vaccination [59, 60]. There were evidences that individuals at high-risk have greater acceptance of the vaccine [59]. Our results indicated it is essential to improve vaccine coverage for these high-risk individuals as soon as possible to prevent a resurgence.

There are some limitations of the current study. Our model did not consider the characteristics of the population, such as age, sex, and occupation. A heterogeneous population might influence vaccine coverage. Thus, a more sophisticated model, such as an agent-based model, is more suited for addressing the issue of population heterogeneity.

## Conclusions

Our study estimated that vaccine coverage of 50.4% was needed before NPIs can be fully relaxed. As vaccine coverage increases, the NPIs can be gradually relaxed. Until that threshold is reached, however, strict NPIs are still needed to contain the epidemic. An accelerated vaccination strategy was the most effective measure for preventing a resurgence, followed by providing vaccination to high-risk groups. Targeting of high-risk groups for vaccination may be the best approach if there are insufficient vaccine doses. The more transmissible SARS-CoV-2 variant led to higher resurgence probability, which indicates the importance of accelerated vaccination and achieving the vaccine coverage earlier.

## Supplementary Information

**Additional file 1: Fig S1.** Sensitivity analyses on resurgence threshold. **Fig. S2.** Sensitivity analyses on transmission rate (**a**), vaccination number per year (**b**), vaccination effectiveness rate (**c**), vaccination effectiveness time (**d**) and imported patients per year (**e**). For each plot, each row represents the date to lift NPIs (months, from the day of vaccination) and each column represents resurgence probability. Colors represent different scenarios of every parameter. Horizontal dotted line in each plot represents the threshold of resurgence probability (20%). **Table S1.** Parameter settings for the main analysis.

## Data Availability

The datasets used and/or analyzed during the current study are available from the corresponding author on reasonable request.

## Abbreviations

COVID-19: Coronavirus disease 2019
SARS-CoV-2: Severe acute respiratory syndrome coronavirus 2
NPIs: Non-pharmaceutical interventions
SIRS: Susceptible Infectious Recovered Susceptible
MC simulation: Monte Carlo simulation
95% *CI*: 95% Confidence interval
MDT: Mean detection time
CDC: Centers for Disease Control and Prevention
WHO: World Health Organization
