# Supplementary material for: Vaccination as an alternative to non-drug interventions to prevent local resurgence of COVID-19
Source: Infect Dis Poverty. 2022 Mar 26;11:36. doi: 10.1186/s40249-022-00960-6 (PMC8959078; doi:10.1186/s40249-022-00960-6)
Supplement: Supplementary file 1 — Additional file 1: Fig S1. Sensitivity analyses on resurgence threshold. Fig. S2. Sensitivity analyses on transmission rate (a), vaccination number per year (b), vaccination effectiveness rate (c), vaccination effectiveness time (d) and imported patients per year (e). For each plot, each row represents the date to lift NPIs (months, from the day of vaccination) and each column represents resurgence probability. Colors represent different scenarios of every parameter. Horizontal dotted line in each plot represents the threshold of resurgence probability (20%). Table S1. Parameter settings for the main analysis. [file 40249_2022_960_MOESM1_ESM.docx]

Supplementary Materials for

Vaccination as an alternative to non-drug interventions to prevent local resurgence of COVID-19

Jinhua Pan^¶^, Wenlong Zhu^¶^, Jie Tian, Zhixi Liu, Ao Xu, Ye Yao, Weibing Wang

Correspondence to: Dr. Ye Yao (yyao@fudan.edu.cn) and Dr. Weibing Wang (wwb@fudan.edu.cn)

¶ These authors contributed equally to this work.

**This PDF file includes:**

Fig. S1

Fig. S2

Table S1


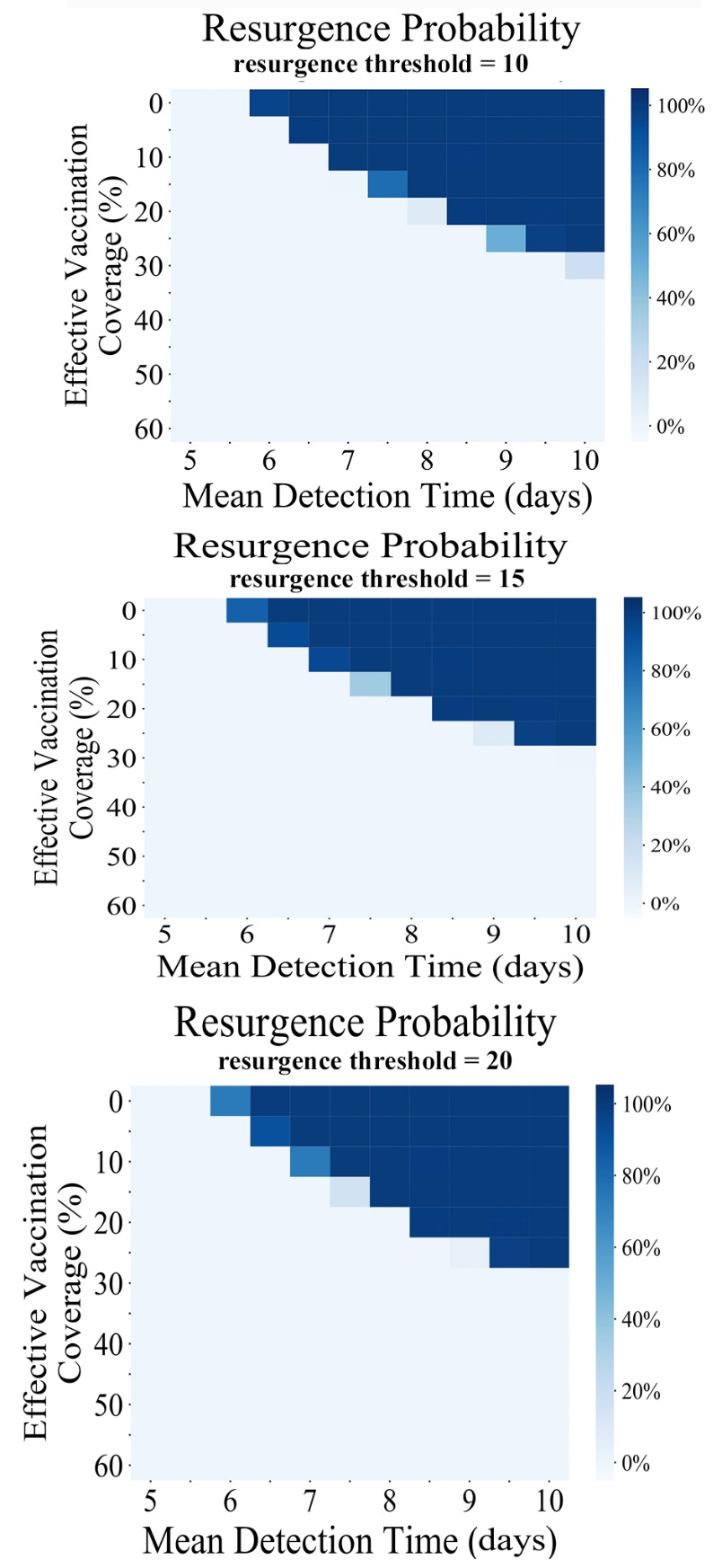


Fig S1. Sensitivity analyses on resurgence threshold.


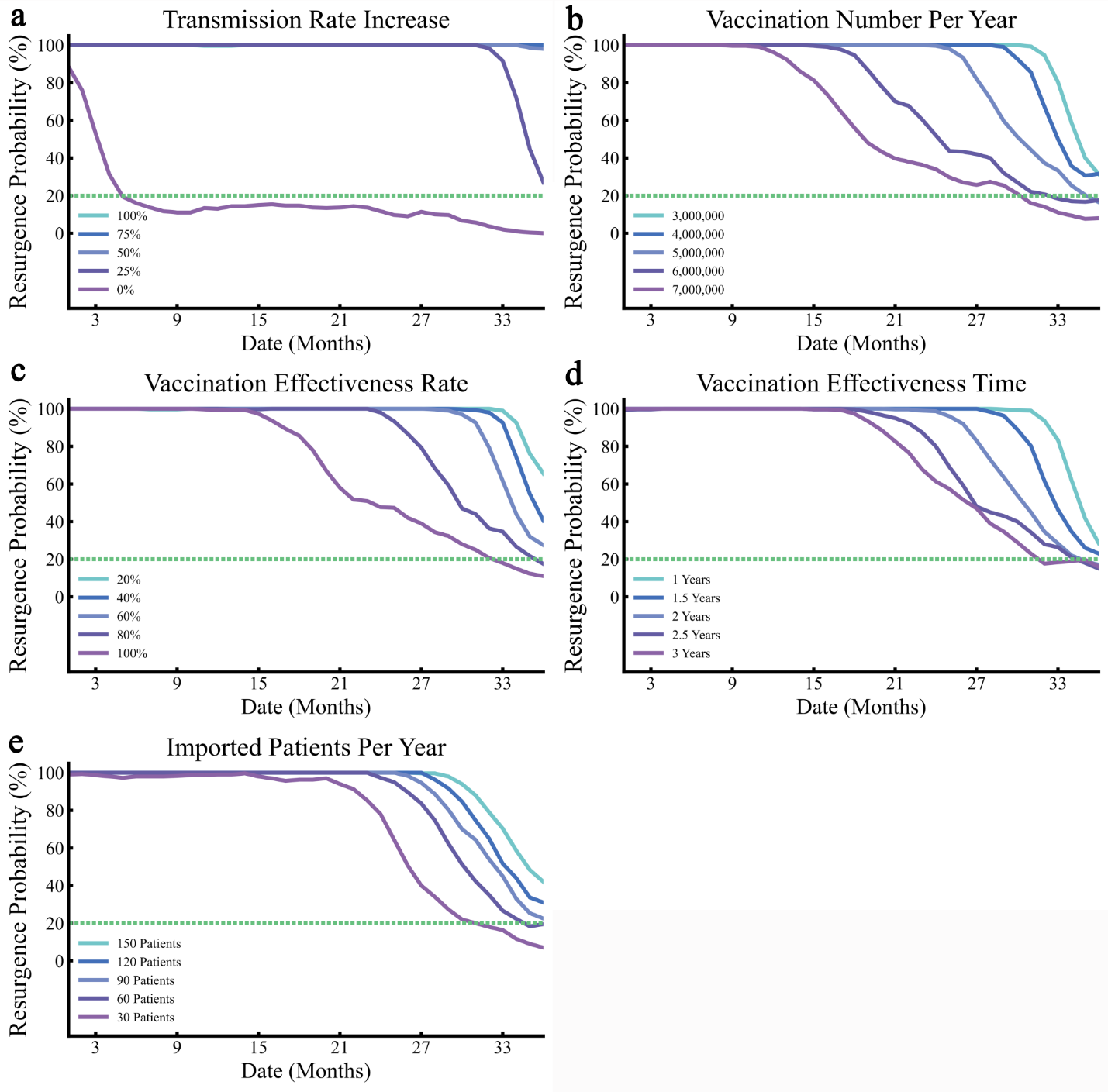
Fig. S2.

**Sensitivity analyses on transmission rate (a), vaccination number per year (b), vaccination effectiveness rate (c), vaccination effectiveness time (d) and imported patients per year (e).** For each plot, each row represents the date to lift NPIs (months, from the day of vaccination) and each column represents resurgence probability. Colors represent different scenarios of every parameter. Horizontal dotted line in each plot represents the threshold of resurgence probability (20%).

Table S1.

Parameter settings for the main analysis.

| Parameter |  | Description |  | Value / Initial State | Unit |  | Range |
| --- | --- | --- | --- | --- | --- | --- | --- |
| β |  | Transmission rate of COVID-19 |  | 0.16-0.34 | NA |  | Stochastic Process |
| 1/γ_1_ |  | Time of patients in I compartment |  | 14 | Day |  | 10% perturbation |
| 1/γ_2_ |  | Time of patients in Q compartment |  | 14 | Day |  | 10% perturbation |
| b |  | Adverse reaction rate |  | 0.0002 | NA |  | 10% perturbation |
| e |  | Vaccine effectiveness |  | 0.7934 | NA |  | 10% perturbation |
| V |  | Daily vaccination number |  | Slow Scenarios:  5,000,000 per year  Accelerated Scenarios:  16,000,000 first year | Daily vaccination number |  | 10% perturbation |
| σ |  | Decline rate of vaccine efficacy |  | 1/730 | NA |  | 10% perturbation |
| A |  | Sporadic foreign imported COVID-19 cases |  | Frequency: 20/120 | NA |  | Poisson process |
| C |  | Number of patients selected randomly from a discrete uniform distribution each time |  | 1~7 | People |  |  |
| 1/h |  | Mean detection time |  | 5~10 | Day |  | 10% perturbation |
| d_1_ |  | Case fatality rates of I compartment |  | 0.005/21 | NA |  | 10% perturbation |
| d_2_ |  | Case fatality rates of Q compartment |  | 0.005/21 | NA |  | 10% perturbation |
| N |  | Total population |  | 21,540,000 | People |  |  |
| S |  | Susceptible people |  | 21,540,000 | People |  |  |
| R |  | Recovered people |  | 0 | People |  |  |
